# Factors influencing poor communication function in children with cerebral palsy: a multivariate analysis

**DOI:** 10.3389/fneur.2026.1842400

**Published:** 2026-06-19

**Authors:** Lingxiao Tong, Baofeng Yan, Songhai Biedelehan, Kaidiriye Abolaiti, Xinping Luan, Aikebaier Halike

**Affiliations:** Cerebral Palsy Center in Neurosurgery, Second Affiliated Hospital of Xinjiang Medical University, Urumqi, Xinjiang, China

**Keywords:** cerebral palsy, communication function, communication function classification system, influencing factors, prognosis

## Abstract

**Objective:**

This study investigates the factors influencing poor communication function outcomes in children with cerebral palsy.

**Methods:**

This retrospective study included children with CP treated at the Department of Neurosurgery, Second Affiliated Hospital of Xinjiang Medical University, between January 2017 and December 2022. Communication function was assessed using the Communication Function Classification System (CFCS), with CFCS levels I–III defined as good communication function and levels IV–V as poor communication function. Demographic, perinatal, maternal, feeding-related, and family-history variables were extracted from clinical records. Univariate analysis and multivariable logistic regression were performed to identify factors associated with poor communication outcomes.

**Results:**

A total of 402 children with CP were included in the analysis. Poor communication function was observed in 156 children. In the multivariable logistic regression analysis, younger age, low birth weight, maternal smoking during pregnancy, non-breastfeeding, and a positive family history were independently associated with poor communication outcomes.

**Conclusion:**

In conclusion, younger age, low birth weight, maternal smoking during pregnancy, non-breastfeeding, and a positive family history were independently associated with poor communication outcomes in children with CP.

## 1 Introduction

Cerebral palsy (CP) is the most prevalent motor dysfunction syndrome in childhood, with a global prevalence of approximately 2–3.5 per 1,000 live births and more than 6 million existing cases in mainland China (1). Despite substantial advancements in rehabilitation medicine that have significantly improved motor function, communication dysfunction remains a major barrier to societal integration for affected children. It is estimated that around 50% of children with CP face communication deficits, with communication disorders affecting up to 60% of children aged 5–6 years (2). Research indicates that 21–36% of children with CP suffer from impaired speech intelligibility due to dysarthria, while 19–32% experience a complete loss of functional speaking ability (3). This communication impairment directly affects the ability of children to express basic needs, leading to a significant reduction in social participation and creating a cycle of ‘dysfunction-social isolation-developmental delay’ (4). Although augmentative and alternative communication (AAC) technology has proven effective in enhancing interaction for children with severe communication impairments, only 31% of those eligible receive such support in clinical practice (5). This highlights the urgent need for early identification of high-risk groups and accurate prognostic evaluations.

Qualitative and parent-perspective studies have shown that communication difficulties in children with CP extend beyond speech impairment and may affect daily participation, social interaction, family communication, AAC use, and access to appropriate support (6–9). However, previous studies have mainly described communication profiles, participation restrictions, or intervention needs, while relatively few have systematically examined independent clinical and perinatal factors associated with poor communication outcomes (6–9). Therefore, further identification of early-life factors related to poor communication function may help improve risk screening and individualized rehabilitation planning in children with CP. To address this, the present study examined a cohort of 402 children diagnosed with CP, who were admitted to the Second Affiliated Hospital of Xinjiang Medical University from 2017 to 2022. The primary objective was to quantify communication function outcomes using the CFCS grading system. The secondary aim was to systematically analyze the impact of multidimensional factors, including perinatal characteristics, genetic background, and feeding style, on communication prognosis. Ultimately, the study seeks to identify independent risk factors for communication dysfunction in children with CP, providing evidence-based support for early screening of high-risk groups and the development of individualized communication rehabilitation strategies, with significant implications for perinatal healthcare interventions.

## 2 Materials and methods

### 2.1 Study subjects

Patients who attended the cerebral palsy center at the Second Affiliated Hospital of Xinjiang Medical University between January 2017 and December 2022 were included in the study. Inclusion criteria were as follows: (1) diagnosis according to internationally recognized CP criteria (10); (2) age between 2 and 18 years; (3) complete birth, pregnancy, and perinatal records; and (4) comprehensive assessment of communication function. The age range of 2–18 years was selected to reflect the broad pediatric population of children with CP in clinical practice, from early childhood to adolescence. Because communication function may change with development, age was included as a candidate variable in the statistical analysis. Exclusion criteria were as follows: (1) children with behavioral abnormalities, severe intellectual disability (IQ score <50), or epilepsy; (2) other neurological disorders (e.g., hydrocephalus, stroke, meningitis) that may impair communication function; (3) conditions that could cause hearing impairments; and (4) CP who had received relevant treatments prior to admission.

The final cohort consisted of 402 children with CP treated at the Department of Neurosurgery, Second Affiliated Hospital of Xinjiang Medical University. The study was approved by the hospital’s Medical Ethics Committee (Approval No. 20211012-14C), and informed consent was obtained from patients and their families for data use.

### 2.2 Evaluation of communication function

The Communication Function Classification System (CFCS) was used to standardize the communication function assessment in this study (11). The CFCS evaluates daily communication performance, including speech, gestures, and assistive technology use, categorizing communication function into five levels: Level I: effective use of communication or assistive devices for complex conversations (e.g., initiating topics, understanding abstract concepts); Level II: effective communication in familiar environments but requiring adaptation in more complex situations; Level III: simplification and repetition of communication or assistive devices for basic communication; Level IV: communication limited to expressing needs through assistive devices or simple gestures; Level V: no functional oral communication, dependent on others to interpret non-verbal cues (e.g., facial expressions, body movements).

Assessments were conducted independently by two neurodevelopmental specialists (associate physician or above) who underwent uniform training and passed a consistency test (Kappa = 0.82) before evaluation. The assessment included spontaneous communication proficiency in familial, medical, and social contexts. Each session lasted 30–45 min, including parent interviews and behavioral observations. CFCS grading results were reviewed by both physicians, with a third senior physician serving as an arbitrator in case of disagreement. According to the CFCS classification, children in CFCS levels 1–3 were categorized as having good communication function, capable of effective communication, though some required more time or familiar objects. Children in CFCS levels 4–5 were classified as having poor communication function, facing significant communication challenges despite the use of auxiliary aids or familiar communication partners (12).

### 2.3 Data collection and variable definition

The selected variables were chosen *a priori* because they are routinely available in clinical records and may reflect biological and environmental influences on early neurodevelopment that are relevant to later communication outcomes in children with CP. Data were collected using a standardized case report form designed for this study. The CFCS was used only to assess communication function, whereas demographic, perinatal, maternal, feeding-related, and family-history variables were extracted from medical records and parent-reported information when necessary. Data collected for children with CP included sex, age, gestational age, birth weight, breastfeeding status, neonatal asphyxia, communication function, and family history. Maternal factors included age at conception, smoking during pregnancy, infection during pregnancy, alcohol consumption during pregnancy, and malnutrition during pregnancy. Paternal age at conception was also recorded.

Operational definitions were as follows: preterm birth was defined as delivery before 37 completed weeks of gestation; low birth weight as birth weight <2,500 g; neonatal asphyxia as a 5-min Apgar score ≤7; smoking during pregnancy as smoking at least one cigarette per day during pregnancy; infection during pregnancy as a hospital-diagnosed maternal infection during pregnancy; breastfeeding as breastfeeding for at least 6 months; and positive family history as CP diagnosed in at least two family members, including the proband.

### 2.4 Statistical analysis

Statistical analyses were performed using IBM SPSS Statistics version 25.0 (IBM Corp., Armonk, NY, USA). Continuous variables were tested for normality. Normally distributed continuous variables were expressed as mean ± standard deviation (SD) and compared using the independent-samples t test or one-way analysis of variance, as appropriate. Non-normally distributed continuous variables were presented as median (interquartile range) and compared using the Mann–Whitney U test. Categorical variables were expressed as frequencies and percentages and compared using the chi-square test or Fisher’s exact test, as appropriate. To control for potential confounding, variables with *p* < 0.10 in the univariate analysis, together with clinically relevant perinatal factors identified *a priori*, were entered into a multivariable binary logistic regression model. Because communication outcome was categorized as CFCS I–III versus CFCS IV–V, binary logistic regression was used to estimate adjusted odds ratios (ORs) and 95% confidence intervals (CIs). A two-sided *p*-value < 0.05 was considered statistically significant.

## 3 Results

### 3.1 Baseline characteristics

The study included 402 patients diagnosed with CP, with 240 males (59.7%) and 162 females (40.3%). The median age of the patients was 7 years (interquartile range: 5–9 years). The most common type of CP was spastic CP (208 cases, 51.7%), followed by mixed CP (177 cases, 44.0%) and dyskinetic cerebral palsy (17 cases, 4.2%). According to the Communication Functional Classification System (CFCS), the distribution of patients’ functional levels was as follows: Level I (74 cases, 18.4%), Level II (99 cases, 24.6%), Level III (73 cases, 18.2%), Level IV (97 cases, 24.1%), and Level V (59 cases, 14.7%) (Table 1).

**Table 1:** Baseline data.

Characteristics	All (*n* = 402 cases)
Gender (numbers)	
Male	240
Female	162
Age (Years)	7 (5, 9)
Cerebral palsy classification (numbers)	
Mixed cerebral palsy	177
Spastic cerebral palsy	208
Athetoid cerebral palsy	17
CFCS (numbers)	
Level I	74
Level II	99
Level III	73
Level IV	97
Level V	59

### 3.2 Univariate analysis

Univariate analysis comparing the CFCS I-III group (246 cases) and the CFCS IV-V group (156 cases) revealed no significant difference in gender distribution (*χ*^2^ = 0.652, *p* = 0.460). The CFCS IV-V group had a significantly lower median age (5.5 years vs. 8 years, *Z* = 4.927, *p* < 0.001), but no significant differences were found in the parental age (both *p* > 0.05).

In terms of perinatal factors, significant associations were observed between the CFCS IV-V group and abnormal birth weight (*χ*^2^ = 4.316, *p* = 0.018), not being breastfed (*χ*^2^ = 5.292, *p* = 0.015), and smoking during pregnancy (*χ*^2^ = 5.687, *p* = 0.017). However, preterm labor and mode of delivery had no significant effects (all *p* > 0.05).

Regarding genetic and environmental factors, a family history of CP was more prevalent in the CFCS IV-V group (*χ*^2^ = 4.099, *p* = 0.043), while alcohol consumption during pregnancy, infections, and malnutrition did not show significant associations (all *p* > 0.05) (Table 2).

**Table 2:** Univariate analysis of factors influencing communication function outcome.

Variables	CFCS I-III group (*n* = 246)	CFCS IV-V group (*n* = 156)	*χ* ^2^ */Z*	*p*-value
Gender				
Male (= 0)	143	97		
Female (= 1)	103	59	0.652	0.460
Age	8 (5, 10)	5.5 (4, 8.75)	4.927	<0.001
Age of mother	25 (22, 28)	25 (21, 27)	0.343	0.732
Age of father	25 (22, 29)	26 (23, 28)	0.290	0.772
Preterm birth (< 37 weeks)				
Yes	85	44		
No	161	112	1.765	0.184
Birth weight (< 2,500 g)				
Yes	57	54		
No	189	102	6.256	0.012
Breast milk (≥ 6 months)				
Yes	141	70		
No	105	86	5.292	0.015
Smoking during pregnancy				
Yes	34	36		
No	212	120	5.687	0.017
Drinking during pregnancy				
Yes	48	31		
No	198	125	0.008	0.930
Neonatal asphyxia				
Yes	119	61		
No	127	95	3.319	0.069
Delivery mode				
Vaginal delivery	157	97		
Cesarean section	89	59	0.111	0.739
Infection during pregnancy				
Yes	34	16		
No	212	140	1.114	0.291
Malnutrition during gestation				
Yes	33	20		
No	213	136	0.029	0.854
Family genetic history				
Yes	10	14		
No	236	142	4.099	0.043

### 3.3 Multivariate logistic regression

Continuous variables were analyzed using receiver operating characteristic (ROC) curves to determine optimal cut-off values; for age, the optimal cut-off was 5.5 years. Prior to multivariable logistic regression, the independent variables included in the model were assessed for multicollinearity, and no significant multicollinearity was detected (Table 3). Model calibration was evaluated using the Hosmer-Lemeshow goodness-of-fit test, which was non-significant (*χ*^2^ = 2.861, *p* = 0.943), indicating an acceptable model fit. After adjustment for potential confounders, multivariable logistic regression identified several factors significantly associated with poor communication outcome (CFCS IV-V). Age >5.5 years (odds ratio [OR] = 0.398, 95% confidence interval [CI] = 0.276–0.574, *p* < 0.001) and breastfeeding (OR = 0.612, 95% CI = 0.400–0.937, *p* = 0.024) were associated with a lower risk of poor communication outcome. In contrast, abnormal birth weight (OR = 1.716, 95% CI = 1.012–2.908, *p* = 0.045), smoking during pregnancy (OR = 1.804, 95% CI = 1.037–3.139, *p* = 0.037), and a positive family history (OR = 2.543, 95% CI = 1.060–6.102, p = 0.037) were independently associated with an increased risk of poor communication outcome. Preterm birth and neonatal asphyxia were not independently associated with communication outcome in the adjusted model (both *p* > 0.05) (Table 4).

**Table 3:** Multicollinearity diagnosis of candidate independent variables.

Factors(self-variable name)	VIF
Age	1.568
Preterm birth (< 37 weeks)	1.037
Birth weight (< 2,500 g)	1.563
Breast milk (≥ 6 months)	1.034
Smoking during pregnancy	1.041
Neonatal asphyxia	1.045
Family genetic history	1.568

**Table 4:** Logistic regression analysis for multiple factors affecting the communication function outcome.

					Exp (*B*) 95% C.I.	
Factors	*B* value	S.E. value	*p*-value	Exp (*B*)	Lower limit	Upper limit
Age	−0.921	0.187	<0.001	0.398	0.276	0.574
Preterm birth	0.125	0.265	0.638	1.113	0.674	1.907
Birth weight	0.540	0.269	0.045	1.716	1.012	2.908
Breast milk	−0.491	0.217	0.024	0.612	0.400	0.937
Smoking during pregnancy	0.590	0.283	0.037	1.804	1.037	3.139
Neonatal asphyxia	−0.116	0.217	0.592	0.890	0.582	1.362
Family genetic history	0.993	0.447	0.037	2.543	1.060	6.102

## 4 Discussion

This retrospective study identified several factors independently associated with poor communication outcomes in children with CP after multivariable adjustment, including younger age, abnormal birth weight, non-breastfeeding, maternal smoking during pregnancy, and a positive family history. These findings extend previous research by highlighting not only biological and perinatal vulnerability, but also modifiable early-life factors that may help clinicians identify children at increased risk of communication difficulties at an earlier stage. From a clinical perspective, these results support a more comprehensive risk assessment framework for children with CP that goes beyond motor impairment alone. The multivariable analysis allowed adjustment for important demographic and perinatal factors, thereby reducing the influence of potential confounding on the observed associations.

Extensive research has long established low birth weight as a significant risk factor for neurodevelopmental disorders and CP in children (13). The present study’s findings further corroborate this assertion. A large retrospective study conducted in the United States on the impact of birth weight on developmental disorders found that low birth weight was linked to later development of conduct disorders, attention deficit hyperactivity disorder, learning disabilities, and other developmental delays. This aligns with our findings that low birth weight adversely affects communication function. Similarly, a study in Korea on the long-term neurodevelopmental outcomes of low birth weight infants highlighted that, with advances in medical care, many low birth weight infants now require communication support, further suggesting that low birth weight significantly impacts communication function (14). The current study demonstrates that the likelihood of poor communication function outcomes in low birth weight children is 1.7 times higher than in children with normal birth weight. It is well established that risk factors for CP are linked to white matter damage and perilesional softening of white matter (PVL), and that communication pathways and networks are intricately connected to white matter (15). A machine learning study aimed at predicting communication development in low birth weight infants found that the microstructure of white matter in these infants differed significantly from that of full-term infants (16). This suggests that communication dysfunction in low birth weight infants is primarily due to white matter damage, which aligns with our findings. From a clinical perspective, children with CP and a history of low birth weight may benefit from earlier and more frequent communication screening, with timely referral for speech-language assessment and intervention when early delays are detected.

Previous studies have demonstrated that smoking during pregnancy negatively impacts the neurological development of offspring and is a significant risk factor for neurodevelopmental disorders (17). A large-scale retrospective cohort study involving 81,066 mothers in Denmark found that smoking during pregnancy increases the risk of congenital CP in offspring, confirming its role as a major risk factor. The present study found that the prevalence of female smoking within the families of children with CP was 27%, notably higher than in the general population, suggesting that smoking may contribute to the development of CP. The study also showed that children with CP from families with a history of maternal smoking during pregnancy had a 1.8 times higher risk of poor communication function compared to those from families without such a history. Animal studies have indicated that nicotine exposure during pregnancy reduces uterine blood flow and disrupts the differentiation of fetal neural cells. Additionally, smoking during pregnancy has been shown to adversely affect the developing white matter in the brains of adolescents (18). Given that white matter is closely associated with communication function, these findings suggest that smoking during pregnancy not only increases the risk of CP but also potentially damages the child’s white matter, contributing to poor communication outcomes. These findings support the importance of strengthening smoking cessation counseling during pregnancy and incorporating prenatal exposure history into the routine risk assessment of children with CP.

CP is widely recognized as a heterogeneous neurodevelopmental disorder, with multiple etiologies identified through large-scale epidemiological studies. However, the specific pathogenesis remains undefined (19). With the advancement of genetic technology and the discovery of various genes implicated in CP, the role of family history has become increasingly emphasized (20). A Norwegian retrospective study involving 200,000 individuals highlighted the significance of familial risk, even after adjusting for key factors such as prematurity. This study confirmed that family history remains a significant risk factor for the development of CP, a finding corroborated by our research. The study further revealed that patients with a familial history of CP have a 2.5 times higher risk of poor communication function outcomes compared to children without such a history. Additionally, the prevalence of CP in children with a family history is 2.5 times higher than in those without a family history, supporting the hypothesis of genetic involvement. Furthermore, certain neurological conditions, including autism spectrum disorders and schizophrenia, have been associated with varying degrees of impairment in communication-related brain functions. Similar alterations have been observed in the brains of affected individuals’ relatives and offspring (21). Although family history cannot establish a direct genetic mechanism in the present study, it may help identify a subgroup of children who warrant closer developmental surveillance and, where appropriate, genetic evaluation or counseling.

Advancements in healthcare have led to an increased emphasis on breastfeeding for newborns, as it stimulates neuronal tissue growth compared to traditional formula feeding (22). Active substances in breast milk, including non-coding RNAs, stem cells, and microbiomes, have the ability to cross the blood–brain barrier and interact with specific genes that influence brain development (23). This suggests that these components play a role in the growth and development of the nervous system. A significant body of research has demonstrated the critical role of breastfeeding in children born with low birth weight. Two multicenter randomized controlled trials have highlighted the impact of breastfeeding on growth and developmental outcomes in low birth weight neonates, indicating that breastfeeding supports cognitive development in these children (24, 25). The present study found that children with CP who were breastfed had an OR of 0.612 for poor communication outcomes, indicating that non-breastfed children with CP were 1.6 times more likely to experience poor communication outcomes compared to their breastfed counterparts. Similar findings have been reported in previous studies. For example, a study investigating the modulation of intestinal flora through breastfeeding found it beneficial for communication and cognitive development in infants (26). Another study exploring the relationship between breastfeeding and speech development in children with CP, using the Viking Language Scale score, found that breastfeeding contributed to improved communication function (27). In professional practice, breastfeeding history may serve as a simple and accessible marker in early developmental follow-up. Families of non-breastfed children with CP may require closer monitoring of communication development and earlier caregiver-mediated intervention.

The present study also revealed that children with CP who were older than 5.5 years demonstrated superior communication outcomes compared to those who had not yet reached this age. A related study on age and neurodevelopment suggested an upward trend in reading, comprehension, and communication skills with increasing age (28). The concept of brain plasticity, particularly in response to pre- and postnatal central nervous system injuries, reinforces the idea that impaired functions can improve as the child develops (29). A longitudinal cohort study from the Dutch Pediatric Rehabilitation Study examined the developmental trajectories of communication and social interaction in 421 children with CP over 13 years. The study concluded that the developmental curves of children with CP without intellectual disability were similar to those of typically developing children, and even children with intellectual disability showed continued improvement in development over time (30). This study found that older children with cerebral palsy had better communication skills. However, this association should be interpreted with caution. Communication function is a dynamic process influenced by multiple factors, including early biological and perinatal factors, as well as postnatal rehabilitation, educational experience, interpersonal interaction and environmental support. Therefore, the better performance of older children in the CFCS may reflect the opportunities they have had to train and communicate, rather than being a direct effect of age itself. As this was a retrospective cross-sectional study, it was not possible to adjust for these postnatal factors that might influence the observed association. Further research is needed to explore the relationship between age and communication function.

The lack of independent association between prematurity or neonatal asphyxia and communication outcome in the adjusted model should be interpreted cautiously. These factors may still contribute indirectly through broader neurodevelopmental pathways, but their effects may have been attenuated by sample size, variable categorization, or residual confounding (31). However, the present study did not find a significant effect of these factors on communication function outcomes in children with CP. One potential explanation is that the underlying pathophysiology of CP remains incompletely understood, and these factors may not directly influence communication function. Another possibility is that the study’s focus on a specific subset of preterm births and neonatal asphyxia may have influenced the results. Future research should clarify the impact of neonatal asphyxia and prematurity on communication function in children with CP.

This retrospective study identified several factors associated with poor communication outcomes in children with CP, providing additional evidence for communication-focused risk assessment in this population. However, several limitations should be acknowledged. First, this was a single center retrospective study with a relatively small sample size, which may have introduced selection bias and limited the generalizability of the findings. In addition, children with severe intellectual disability were excluded, potentially resulting in underrepresentation of those with more severe communication difficulties. Second, although family history and birth weight remained statistically significant in the multivariable analysis, the lower bounds of their 95% confidence intervals were close to 1.0, indicating limited precision of these effect estimates. Third, residual confounding cannot be excluded because some relevant variables, such as motor severity, cognitive status, socioeconomic background, and rehabilitation intensity, were not available in this retrospective dataset. Future multicenter prospective studies with larger samples are needed to confirm these findings and to further clarify how perinatal, familial, and postnatal factors jointly influence communication development in children with CP.

The present findings have several implications for professional practice. First, communication risk screening in children with CP should not rely solely on motor severity but should also consider perinatal and family-related factors such as birth weight, prenatal smoking exposure, breastfeeding history, and family history. Second, children with multiple risk factors may benefit from earlier referral for communication outcomes assessment, caregiver training, and augmentative and alternative communication planning. Third, the findings support greater collaboration among obstetric, neonatal, rehabilitation, and speech-language professionals to promote prenatal risk reduction, early developmental surveillance, and individualized communication intervention.

## 5 Conclusion

In this retrospective study, younger age, low birth weight, maternal smoking during pregnancy, non-breastfeeding, and a positive family history were independently associated with poor communication outcomes in children with CP. These findings may provide preliminary evidence for early risk identification and communication-focused developmental surveillance. Nevertheless, because of the observational design of the present study, stronger clinical recommendations regarding prevention systems, long-term tracking, and targeted rehabilitation should be interpreted with caution and require further validation in prospective studies.

## Data Availability

The datasets presented in this article are not publicly available as they contain sensitive patient data. Requests to access the datasets should be directed to akbarhalik@sina.com.

## References

[1] Oskoui M Coutinho F Dykeman J Jetté N Pringsheim T. An update on the prevalence of cerebral palsy: a systematic review and meta-analysis. Dev Med Child Neurol. (2013) 55:509–19. doi: 10.1111/dmcn.12080, 23346889

[2] Mei C Reilly S Reddihough D Mensah F Pennington L Morgan A. Language outcomes of children with cerebral palsy aged 5 years and 6 years: a population-based study. Dev Med Child Neurol. (2016) 58:605–11. doi: 10.1111/dmcn.12957, 26566585

[3] Mei C Reilly S Bickerton M Mensah F Turner S Kumaranayagam D . Speech in children with cerebral palsy. Dev Med Child Neurol. (2020) 62:1374–82. doi: 10.1111/dmcn.14592, 32588921

[4] Alghamdi MS Chiarello LA Palisano RJ McCoy SW. Understanding participation of children with cerebral palsy in family and recreational activities. Res Dev Disabil. (2017) 69:96–104. doi: 10.1016/j.ridd.2017.07.006, 28843215

[5] Lillehaug HA Klevberg GL Stadskleiv K. Provision of augmentative and alternative communication interventions to Norwegian preschool children with cerebral palsy: are the right children receiving interventions? Augment Alternat Commun. (2023) 39:219–29. doi: 10.1080/07434618.2023.2212068 37212772

[6] Choi JY Park J Choi YS Goh YR Park ES. Functional communication profiles in children with cerebral palsy in relation to gross motor function and manual and intellectual ability. Yonsei Med J. (2018) 59:677–85. doi: 10.3349/ymj.2018.59.5.677, 29869466 PMC5990683

[7] Mei C Reilly S Reddihough D Mensah F Green J Pennington L . Activities and participation of children with cerebral palsy: parent perspectives. Disabil Rehabil. (2015) 37:2164–73. doi: 10.3109/09638288.2014.999164 25586796

[8] Smith AL Hustad KC. AAC and early intervention for children with cerebral palsy: parent perceptions and child risk factors. Augment Alternat Commun. (2015) 31:336–50. doi: 10.3109/07434618.2015.1084373 PMC4628599 26401966

[9] O'Neill T Wilkinson KM. Preliminary investigation of the perspectives of parents of children with cerebral palsy on the supports, challenges, and realities of integrating augmentative and alternative communication into everyday life. Am J Speech Lang Pathol. (2020) 29:238–54. doi: 10.1044/2019_AJSLP-19-00103, 31961702

[10] Bax M Goldstein M Rosenbaum P Leviton A Paneth N Dan B . Proposed definition and classification of cerebral palsy, April 2005. Dev Med Child Neurol. (2005) 47:571–6. doi: 10.1017/S001216220500112X, 16108461

[11] Piscitelli D Ferrarello F Ugolini A Verola S Pellicciari L. Measurement properties of the gross motor function classification system, Gross Motor Function Classification System-Expanded & Revised, manual ability classification system, and communication function classification system in cerebral palsy: a systematic review with meta-analysis. Dev Med Child Neurol. (2021) 63:1251–61. doi: 10.1111/dmcn.14910, 34028793

[12] Wu J Bai C Yan B Mutalifu N Guan Q Li J . Development and validation of a predictive model for poor prognosis of communication disorders in children with cerebral palsy after cervical perivascular sympathectomy. Neurosurg Rev. (2024) 47:142. doi: 10.1007/s10143-024-02380-6, 38587684 PMC11001727

[13] Wu J Yan B Mutalifu N Guan Q Bai C Li J . Efficacy and influencing factors of cervical perivascular sympathectomy in children with cerebral palsy. Child's Nerv Syst. (2024) 40:1137–45. doi: 10.1007/s00381-023-06191-w, 37870563 PMC10972969

[14] O'Shea TM Dammann O. Antecedents of cerebral palsy in very low-birth weight infants. Clin Perinatol. (2000) 27:285–302. doi: 10.1016/S0095-5108(05)70022-1, 10863651

[15] Agut T Alarcon A Cabañas F Bartocci M Martinez-Biarge M Horsch S. Preterm white matter injury: ultrasound diagnosis and classification. Pediatr Res. (2020) 87:37–49. doi: 10.1038/s41390-020-0781-1, 32218534 PMC7098888

[16] Valavani E Blesa M Galdi P Sullivan G Dean B Cruickshank H . Language function following preterm birth: prediction using machine learning. Pediatr Res. (2022) 92:480–9. doi: 10.1038/s41390-021-01779-x, 34635792 PMC8503721

[17] Li Q Cai X Zhou H Ma D Li N. Maternal smoking cessation in the first trimester still poses an increased risk of attention-deficit/hyperactivity disorder and learning disability in offspring. Front Public Health. (2024) 12:1386137. doi: 10.3389/fpubh.2024.1386137, 39081356 PMC11286595

[18] Bednarczuk N Milner A Greenough A. The role of maternal smoking in sudden Fetal and infant death pathogenesis. Front Neurol. (2020) 11:586068. doi: 10.3389/fneur.2020.586068, 33193050 PMC7644853

[19] Korzeniewski SJ Slaughter J Lenski M Haak P Paneth N. The complex aetiology of cerebral palsy. Nat Rev Neurol. (2018) 14:528–43. doi: 10.1038/s41582-018-0043-6, 30104744

[20] MacLennan AH Thompson SC Gecz J. Cerebral palsy: causes, pathways, and the role of genetic variants. Am J Obstet Gynecol. (2015) 213:779–88. doi: 10.1016/j.ajog.2015.05.034, 26003063

[21] Tollånes MC Wilcox AJ Lie RT Moster D. Familial risk of cerebral palsy: population based cohort study. BMJ (Clinical research ed). (2014) 349:g4294. doi: 10.1136/bmj.g4294, 25028249 PMC4099475

[22] Kaps J Georgieva VS Oberholz L Kribs A Brachvogel B Keller T. Human preterm colostrum stimulates outgrowth in neurogenic tissue. Pediatr Res. (2023) 94:1906–10. doi: 10.1038/s41390-023-02721-z, 37433903 PMC10665184

[23] Gialeli G Panagopoulou O Liosis G Siahanidou T. Potential epigenetic effects of human Milk on infants' neurodevelopment. Nutrients. (2023) 15:3614. doi: 10.3390/nu15163614, 37630804 PMC10460013

[24] Vohr BR Poindexter BB Dusick AM McKinley LT Higgins RD Langer JC . Persistent beneficial effects of breast milk ingested in the neonatal intensive care unit on outcomes of extremely low birth weight infants at 30 months of age. Pediatrics. (2007) 120:e953–9. doi: 10.1542/peds.2006-3227, 17908750

[25] Vohr BR Poindexter BB Dusick AM McKinley LT Wright LL Langer JC . Beneficial effects of breast milk in the neonatal intensive care unit on the developmental outcome of extremely low birth weight infants at 18 months of age. Pediatrics. (2006) 118:e115–23. doi: 10.1542/peds.2005-2382 16818526

[26] Guo S Huang K Liu R Sun J Yin C. Regulation of gut microbiota through breast Milk feeding benefits language and cognitive development of preterm toddlers. Microorganisms. (2023) 11:866. doi: 10.3390/microorganisms11040866, 37110289 PMC10146954

[27] Kaya Özçora GD. The relationship between breast milk intake and speech in children with cerebral palsy. Turk J Med Sci. (2021) 51:1809–13. doi: 10.3906/sag-2011-43, 33819974 PMC8569807

[28] Liu X Zhang L Yu S Bai Z Qi T Mao H . The effects of age and Reading experience on the lifespan neurodevelopment for Reading comprehension. J Cogn Neurosci. (2024) 36:239–60. doi: 10.1162/jocn_a_02086 38010312

[29] Ismail FY Fatemi A Johnston MV. Cerebral plasticity: windows of opportunity in the developing brain. Euro J Paediatr Neurol. (2017) 21:23–48. doi: 10.1016/j.ejpn.2016.07.007 27567276

[30] Tan SS van Gorp M Voorman JM Geytenbeek JJ Reinders-Messelink HA Ketelaar M . Development curves of communication and social interaction in individuals with cerebral palsy. Dev Med Child Neurol. (2020) 62:132–9. doi: 10.1111/dmcn.14351, 31541474 PMC6916560

[31] Novak I Morgan C Adde L Blackman J Boyd RN Brunstrom-Hernandez J . Early, accurate diagnosis and early intervention in cerebral palsy: advances in diagnosis and treatment. JAMA Pediatr. (2017) 171:897–907. doi: 10.1001/jamapediatrics.2017.1689, 28715518 PMC9641643

